# (*E*)-1-(4-Chloro­phen­yl)-3-[4-(dimethyl­amino)phen­yl]prop-2-en-1-one

**DOI:** 10.1107/S1600536809007430

**Published:** 2009-03-11

**Authors:** Xinyou Lei, Xiaohua Bai

**Affiliations:** a College of Life Sciences and Chemistry, Tianshui Normal University, Tianshu 741000, People’s Republic of China

## Abstract

The title compound, C_17_H_16_ClNO, was synthesized using a solvent-free method by reaction of 4-(dimethyl­amino)benzaldehyde with 4-chloro­acetophenone and NaOH. The chloro­phenyl ring makes a dihedral angle of 18.1 (3)° with the central propenone unit, while the (dimethyl­amino)phenyl group is disordered over two orientations of equal occupancies, which make dihedral angles with the central propenone unit of 32.9 (3) and 57.4 (3)°, respectively.

## Related literature

For a related structure, see: Li *et al.* (1992 ▶).
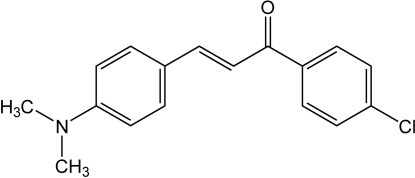


## Experimental

### 

#### Crystal data

C_17_H_16_ClNO
*M* *_r_* = 285.76
Monoclinic, 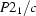

*a* = 16.792 (2) Å
*b* = 14.5602 (16) Å
*c* = 6.1160 (8) Å
β = 98.333 (2)°
*V* = 1479.5 (3) Å^3^
*Z* = 4
Mo *K*α radiation
μ = 0.25 mm^−1^
*T* = 298 K
0.42 × 0.20 × 0.13 mm

#### Data collection

Bruker SMART CCD diffractometer
Absorption correction: multi-scan (*SADABS*; Sheldrick, 2003 ▶) *T*_min_ = 0.901, *T*_max_ = 0.968
7357 measured reflections
2605 independent reflections
1066 reflections with *I* > 2˘*I*)
*R*_int_ = 0.068

#### Refinement

*R*[*F* ^2^ > 2σ(*F* ^2^)] = 0.059
*wR*(*F* ^2^) = 0.170
*S* = 0.90
2605 reflections
237 parameters
4 restraints
H-atom parameters constrained
Δρ_max_ = 0.16 e Å^−3^
Δρ_min_ = −0.12 e Å^−3^

### 

Data collection: *SMART* (Bruker, 1997 ▶); cell refinement: *SAINT* (Bruker, 1997 ▶); data reduction: *SAINT*; program(s) used to solve structure: *SHELXS97* (Sheldrick, 2008 ▶); program(s) used to refine structure: *SHELXL97* (Sheldrick, 2008 ▶); molecular graphics: *SHELXTL* (Sheldrick, 2008 ▶); software used to prepare material for publication: *SHELXTL*.

## Supplementary Material

Crystal structure: contains datablocks I, global. DOI: 10.1107/S1600536809007430/bi2339sup1.cif

Structure factors: contains datablocks I. DOI: 10.1107/S1600536809007430/bi2339Isup2.hkl

Additional supplementary materials:  crystallographic information; 3D view; checkCIF report

## Figures and Tables

**Fig. 1.**
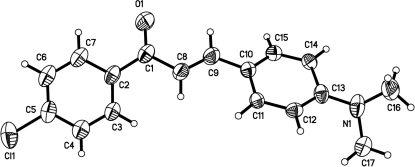
The molecular structure showing 30% probability displacement ellipsoids for non-H atoms.

## supplementary crystallographic information

### Comment

This paper discloses a user-friendly, solvent-free protocol for the synthesis of chalcones, starting from the fragrant aldehydes and fragrant ketones in the presence of NaOH. The method can be considered to be a general route for chalcone synthesis, and the title compound was prepared in this way.

The bond lengths and angles of the molecule (Fig. 1) are normal and comparable to those observed in the related compound (Li *et al.*, 1992). The (dimethylamino)phenyl group exhibits rotational disorder, with one orientation including atoms C10, C11, C12, C13, C14 and C15, and another orientation including C10, C11', C12', C13, C14' and C15'. The refined site occupancy factors for the two orientations is 0.500 (5).

### Experimental

4-(Dimethylamino)benzaldehyde (0.5 mmol) and 4-chloroacetophenone (0.5 mmol), NaOH (0.5 mmol) were mixed in 50 ml flask under solvent-free conditions After stirring for 6 min at 373 K, the mixture was slowly solidified to give the title compound. Recrystallization from ethanol gave a yellow crystalline solid. Elemental analysis calculated: C 71.45, H 5.64, N 4.90%; found: C 71.53, H 5.56, N 4.95%.

### Refinement

All H atoms were positioned geometrically and refined using a riding model with C—H = 0.93–0.96 Å and *U*_iso_(H) = 1.2 or 1.5*U*_eq_(C). The five-membered ring was treated as disordered between two orientations with site occupancy factors refined to 0.500 (5). The bonds N1—C16, N1—C16', N1—C17 and N1—C17' were restrained to be 1.47 (1)Å.

### Crystal data

**Table:**

C_17_H_16_ClNO	*F*(000) = 600
*M**_r_* = 285.76	*D*_x_ = 1.283 Mg m^−^^3^
Monoclinic, *P*2_1_/*c*	Mo *K*α radiation, λ = 0.71073 Å
Hall symbol: -P 2ybc	Cell parameters from 987 reflections
*a* = 16.792 (2) Å	θ = 2.5–19.9°
*b* = 14.5602 (16) Å	µ = 0.25 mm^−^^1^
*c* = 6.1160 (8) Å	*T* = 298 K
β = 98.333 (2)°	Needle, yellow
*V* = 1479.5 (3) Å^3^	0.42 × 0.20 × 0.13 mm
*Z* = 4	

### Data collection

**Table:**

Bruker SMART CCD diffractometer	2605 independent reflections
Radiation source: fine-focus sealed tube	1066 reflections with *I* > 2˘*I*)
graphite	*R*_int_ = 0.068
φ and ω scans	θ_max_ = 25.0°, θ_min_ = 1.9°
Absorption correction: multi-scan (*SADABS*; Sheldrick, 2003)	*h* = −19→19
*T*_min_ = 0.901, *T*_max_ = 0.968	*k* = −17→12
7357 measured reflections	*l* = −7→7

### Refinement

**Table:**

Refinement on *F*^2^	Primary atom site location: structure-invariant direct methods
Least-squares matrix: full	Secondary atom site location: difference Fourier map
*R*[*F*^2^ > 2σ(*F*^2^)] = 0.059	Hydrogen site location: inferred from neighbouring sites
*wR*(*F*^2^) = 0.170	H-atom parameters constrained
*S* = 0.90	*w* = 1/[σ^2^(*F*_o_^2^) + (0.0749*P*)^2^] where *P* = (*F*_o_^2^ + 2*F*_c_^2^)/3
2605 reflections	(Δ/σ)_max_ < 0.001
237 parameters	Δρ_max_ = 0.16 e Å^−^^3^
4 restraints	Δρ_min_ = −0.12 e Å^−^^3^

### Special details

**Table:**

Geometry. All e.s.d.'s (except the e.s.d. in the dihedral angle between two l.s. planes) are estimated using the full covariance matrix. The cell e.s.d.'s are taken into account individually in the estimation of e.s.d.'s in distances, angles and torsion angles; correlations between e.s.d.'s in cell parameters are only used when they are defined by crystal symmetry. An approximate (isotropic) treatment of cell e.s.d.'s is used for estimating e.s.d.'s involving l.s. planes.
Refinement. Refinement of *F*^2^ against ALL reflections. The weighted *R*-factor *wR* and goodness of fit *S* are based on *F*^2^, conventional *R*-factors *R* are based on *F*, with *F* set to zero for negative *F*^2^. The threshold expression of *F*^2^ > σ(*F*^2^) is used only for calculating *R*-factors(gt) *etc*. and is not relevant to the choice of reflections for refinement. *R*-factors based on *F*^2^ are statistically about twice as large as those based on *F*, and *R*- factors based on ALL data will be even larger.

### Fractional atomic coordinates and isotropic or equivalent isotropic displacement parameters (Å^2^)

**Table:**

	*x*	*y*	*z*	*U*_iso_*/*U*_eq_	Occ. (<1)
Cl1	1.12683 (6)	0.14642 (9)	1.2060 (3)	0.1285 (7)	
N1	0.34724 (18)	0.1225 (2)	0.8419 (7)	0.0891 (11)	
O1	0.80650 (17)	0.1009 (2)	0.4377 (6)	0.1225 (12)	
C1	0.8071 (2)	0.1135 (2)	0.6344 (8)	0.0739 (11)	
C2	0.8853 (2)	0.1192 (2)	0.7831 (7)	0.0683 (10)	
C3	0.8921 (2)	0.1538 (2)	0.9959 (7)	0.0742 (11)	
H3	0.8460	0.1712	1.0530	0.089*	
C4	0.9663 (2)	0.1627 (2)	1.1247 (7)	0.0801 (11)	
H4	0.9701	0.1868	1.2666	0.096*	
C5	1.0345 (2)	0.1356 (3)	1.0416 (9)	0.0831 (12)	
C6	1.0292 (3)	0.0990 (3)	0.8348 (9)	0.0889 (13)	
H6	1.0755	0.0792	0.7816	0.107*	
C7	0.9552 (2)	0.0914 (2)	0.7048 (7)	0.0792 (11)	
H7	0.9520	0.0673	0.5629	0.095*	
C8	0.7314 (2)	0.1221 (2)	0.7236 (7)	0.0754 (11)	
H8	0.7343	0.1272	0.8762	0.090*	
C9	0.6615 (2)	0.1233 (3)	0.6088 (7)	0.0872 (12)	
H9	0.6617	0.1225	0.4568	0.105*	
C10	0.5811 (2)	0.1255 (3)	0.6741 (6)	0.0639 (9)	
C11	0.5655 (5)	0.0979 (6)	0.8724 (14)	0.055 (2)	0.500 (5)
H11	0.6076	0.0821	0.9821	0.065*	0.500 (5)
C12	0.4860 (5)	0.0928 (6)	0.9147 (13)	0.059 (2)	0.500 (5)
H12	0.4773	0.0651	1.0463	0.070*	0.500 (5)
C13	0.4234 (2)	0.1232 (2)	0.7863 (7)	0.0639 (10)	
C14	0.4419 (4)	0.1658 (5)	0.5679 (11)	0.057 (2)	0.500 (5)
H14	0.4007	0.1885	0.4641	0.068*	0.500 (5)
C15	0.5208 (4)	0.1695 (5)	0.5295 (12)	0.059 (2)	0.500 (5)
H15	0.5339	0.2012	0.4075	0.070*	0.500 (5)
C16	0.2783 (5)	0.0794 (8)	0.7432 (18)	0.103 (4)	0.500 (5)
H16A	0.2583	0.1102	0.6074	0.154*	0.510 (7)
H16B	0.2383	0.0815	0.8404	0.154*	0.510 (7)
H16C	0.2902	0.0166	0.7128	0.154*	0.510 (7)
C17	0.3364 (5)	0.1683 (7)	1.0661 (13)	0.106 (4)	0.500 (5)
H17A	0.3811	0.1526	1.1758	0.158*	0.510 (7)
H17B	0.2874	0.1466	1.1123	0.158*	0.510 (7)
H17C	0.3338	0.2337	1.0483	0.158*	0.510 (7)
C11'	0.5658 (5)	0.1596 (6)	0.8637 (16)	0.060 (2)	0.500 (5)
H11'	0.6089	0.1842	0.9581	0.073*	0.500 (5)
C12'	0.4923 (4)	0.1618 (6)	0.9327 (12)	0.060 (2)	0.500 (5)
H12'	0.4859	0.1870	1.0688	0.072*	0.500 (5)
C14'	0.4368 (4)	0.0774 (5)	0.6145 (12)	0.059 (2)	0.500 (5)
H14'	0.3949	0.0445	0.5344	0.070*	0.500 (5)
C15'	0.5112 (4)	0.0760 (5)	0.5472 (12)	0.064 (2)	0.500 (5)
H15'	0.5184	0.0436	0.4203	0.076*	0.500 (5)
C16'	0.2804 (4)	0.1498 (7)	0.6472 (14)	0.090 (3)	0.500 (5)
H16D	0.2919	0.2095	0.5931	0.135*	0.490 (7)
H16E	0.2290	0.1509	0.6988	0.135*	0.490 (7)
H16F	0.2791	0.1056	0.5302	0.135*	0.490 (7)
C17'	0.3231 (5)	0.0619 (6)	0.9866 (13)	0.079 (3)	0.500 (5)
H17D	0.3431	0.0019	0.9590	0.119*	0.490 (7)
H17E	0.2654	0.0604	0.9688	0.119*	0.490 (7)
H17F	0.3437	0.0804	1.1347	0.119*	0.490 (7)

### Atomic displacement parameters (Å^2^)

**Table:**

	*U*^11^	*U*^22^	*U*^33^	*U*^12^	*U*^13^	*U*^23^
Cl1	0.0640 (8)	0.1472 (12)	0.1697 (14)	−0.0010 (6)	0.0012 (8)	−0.0022 (9)
N1	0.053 (2)	0.084 (2)	0.132 (3)	0.0039 (18)	0.021 (2)	−0.008 (3)
O1	0.085 (2)	0.211 (4)	0.078 (2)	0.001 (2)	0.0324 (18)	0.006 (2)
C1	0.069 (3)	0.072 (3)	0.087 (3)	−0.0025 (19)	0.033 (2)	0.000 (2)
C2	0.061 (2)	0.061 (2)	0.089 (3)	−0.0045 (18)	0.032 (2)	0.003 (2)
C3	0.059 (3)	0.080 (3)	0.087 (3)	0.0014 (18)	0.024 (2)	−0.003 (2)
C4	0.074 (3)	0.075 (3)	0.094 (3)	−0.001 (2)	0.022 (2)	−0.003 (2)
C5	0.060 (3)	0.074 (3)	0.118 (4)	0.003 (2)	0.023 (3)	0.007 (3)
C6	0.071 (3)	0.084 (3)	0.120 (4)	0.003 (2)	0.043 (3)	−0.005 (3)
C7	0.074 (3)	0.074 (3)	0.097 (3)	0.001 (2)	0.037 (3)	−0.007 (2)
C8	0.062 (2)	0.090 (3)	0.077 (3)	−0.010 (2)	0.021 (2)	−0.015 (2)
C9	0.071 (3)	0.130 (3)	0.063 (3)	0.021 (2)	0.018 (2)	0.019 (2)
C10	0.058 (2)	0.075 (2)	0.059 (3)	0.007 (2)	0.0076 (19)	0.001 (2)
C11	0.050 (5)	0.057 (5)	0.056 (6)	0.007 (4)	0.003 (4)	0.004 (5)
C12	0.069 (6)	0.057 (5)	0.052 (5)	−0.006 (5)	0.013 (4)	0.000 (4)
C13	0.050 (2)	0.054 (2)	0.087 (3)	0.0039 (19)	0.005 (2)	−0.008 (2)
C14	0.057 (5)	0.051 (5)	0.058 (5)	0.002 (3)	0.000 (3)	0.008 (3)
C15	0.066 (5)	0.053 (5)	0.058 (5)	−0.007 (4)	0.012 (4)	0.003 (3)
C16	0.076 (6)	0.114 (8)	0.118 (10)	−0.024 (6)	0.015 (6)	−0.030 (7)
C17	0.071 (6)	0.151 (9)	0.098 (8)	−0.002 (5)	0.025 (5)	−0.002 (6)
C11'	0.053 (5)	0.058 (6)	0.067 (6)	−0.005 (5)	−0.001 (4)	−0.012 (5)
C12'	0.058 (5)	0.072 (6)	0.051 (5)	−0.008 (5)	0.011 (4)	−0.008 (4)
C14'	0.047 (4)	0.060 (6)	0.067 (5)	−0.005 (3)	0.000 (4)	−0.011 (4)
C15'	0.067 (5)	0.064 (6)	0.060 (5)	0.005 (4)	0.008 (4)	−0.006 (4)
C16'	0.049 (5)	0.111 (8)	0.105 (8)	−0.001 (5)	−0.001 (5)	0.014 (6)
C17'	0.076 (5)	0.094 (7)	0.071 (7)	0.011 (5)	0.023 (5)	0.011 (5)

### Geometric parameters (Å, °)

**Table:**

Cl1—C5	1.729 (4)	C11—H11	0.930
N1—C17'	1.353 (7)	C12—C13	1.296 (8)
N1—C13	1.370 (4)	C12—H12	0.930
N1—C16	1.377 (7)	C13—C14'	1.292 (7)
N1—C17	1.559 (7)	C13—C12'	1.468 (8)
N1—C16'	1.565 (7)	C13—C14	1.545 (8)
O1—C1	1.215 (4)	C14—C15	1.379 (8)
C1—C8	1.460 (5)	C14—H14	0.930
C1—C2	1.486 (5)	C15—H15	0.930
C2—C3	1.385 (5)	C16—H16A	0.960
C2—C7	1.391 (4)	C16—H16B	0.960
C3—C4	1.379 (5)	C16—H16C	0.960
C3—H3	0.930	C17—H17A	0.960
C4—C5	1.376 (5)	C17—H17B	0.960
C4—H4	0.930	C17—H17C	0.960
C5—C6	1.364 (5)	C11'—C12'	1.362 (10)
C6—C7	1.380 (5)	C11'—H11'	0.930
C6—H6	0.930	C12'—H12'	0.930
C7—H7	0.930	C14'—C15'	1.372 (9)
C8—C9	1.278 (5)	C14'—H14'	0.930
C8—H8	0.930	C15'—H15'	0.930
C9—C10	1.463 (5)	C16'—H16D	0.960
C9—H9	0.930	C16'—H16E	0.960
C10—C11'	1.320 (9)	C16'—H16F	0.960
C10—C11	1.339 (9)	C17'—H17D	0.960
C10—C15	1.399 (8)	C17'—H17E	0.960
C10—C15'	1.495 (8)	C17'—H17F	0.960
C11—C12	1.398 (10)		
			
C17'—N1—C13	123.1 (5)	N1—C13—C12'	122.2 (5)
C13—N1—C16	130.6 (5)	C12—C13—C14	114.4 (5)
C13—N1—C17	116.6 (4)	N1—C13—C14	122.0 (4)
C16—N1—C17	112.5 (5)	C15—C14—C13	118.8 (6)
C17'—N1—C16'	114.4 (6)	C15—C14—H14	120.6
C13—N1—C16'	113.7 (5)	C13—C14—H14	120.6
O1—C1—C8	120.0 (4)	C14—C15—C10	119.9 (6)
O1—C1—C2	119.6 (3)	C14—C15—H15	120.0
C8—C1—C2	120.4 (4)	C10—C15—H15	120.0
C3—C2—C7	118.0 (4)	N1—C16—H16A	109.5
C3—C2—C1	122.8 (3)	N1—C16—H16B	109.5
C7—C2—C1	119.2 (4)	H16A—C16—H16B	109.5
C4—C3—C2	121.1 (4)	N1—C16—H16C	109.5
C4—C3—H3	119.5	H16A—C16—H16C	109.5
C2—C3—H3	119.5	H16B—C16—H16C	109.5
C5—C4—C3	119.5 (4)	N1—C17—H17A	109.5
C5—C4—H4	120.2	N1—C17—H17B	109.5
C3—C4—H4	120.2	H17A—C17—H17B	109.5
C6—C5—C4	120.6 (4)	N1—C17—H17C	109.5
C6—C5—Cl1	120.5 (3)	H17A—C17—H17C	109.5
C4—C5—Cl1	118.9 (4)	H17B—C17—H17C	109.5
C5—C6—C7	119.8 (4)	C10—C11'—C12'	125.5 (7)
C5—C6—H6	120.1	C10—C11'—H11'	117.2
C7—C6—H6	120.1	C12'—C11'—H11'	117.2
C6—C7—C2	120.9 (4)	C11'—C12'—C13	118.2 (7)
C6—C7—H7	119.6	C11'—C12'—H12'	120.9
C2—C7—H7	119.6	C13—C12'—H12'	120.9
C9—C8—C1	125.2 (4)	C13—C14'—C15'	121.8 (6)
C9—C8—H8	117.4	C13—C14'—H14'	119.1
C1—C8—H8	117.4	C15'—C14'—H14'	119.1
C8—C9—C10	131.4 (4)	C14'—C15'—C10	121.1 (6)
C8—C9—H9	114.3	C14'—C15'—H15'	119.4
C10—C9—H9	114.3	C10—C15'—H15'	119.4
C11—C10—C15	119.4 (5)	N1—C16'—H16D	109.5
C11—C10—C9	123.4 (4)	N1—C16'—H16E	109.5
C15—C10—C9	116.7 (4)	H16D—C16'—H16E	109.5
C9—C10—C15'	122.2 (4)	N1—C16'—H16F	109.5
C10—C11—C12	120.1 (6)	H16D—C16'—H16F	109.5
C10—C11—H11	120.0	H16E—C16'—H16F	109.5
C12—C11—H11	120.0	N1—C17'—H17D	109.5
C13—C12—C11	125.6 (7)	N1—C17'—H17E	109.5
C13—C12—H12	117.2	H17D—C17'—H17E	109.5
C11—C12—H12	117.2	N1—C17'—H17F	109.5
C14'—C13—N1	118.5 (4)	H17D—C17'—H17F	109.5
C12—C13—N1	123.5 (5)	H17E—C17'—H17F	109.5
C14'—C13—C12'	118.6 (5)		
